# Supplementary material for: Are carbon emissions trading and green financial instruments synergistic? -Comprehensive quantitative research based on content analysis
Source: PLoS One. 2024 Mar 7;19(3):e0298601. doi: 10.1371/journal.pone.0298601 (PMC10919601; doi:10.1371/journal.pone.0298601)
Supplement: S1 Data — (ZIP) [file pone.0298601.s001.zip › Data/地级市Z_F工作报告.zip!/╝¬┴╓/═¿╗»╩╨/└┤╫╘╣1⁄2╓┌║┼í╛┬φ┐╦╩2╛▌═°í┐_2015.docx]

各位代表：

　　现在，我代表市政府，向大会作政府工作报告，请予审议，并请市政协各位委员提出意见。

　　一、2014年工作回顾

　　2014年，在省委、省政府和市委的坚强领导下，在市人大、市政协的监督支持下，市政府同全市人民一道，攻坚克难，真抓实干，较好完成了市七届人大二次会议确定的各项任务。

　　——经济运行稳中有进、稳中提质。预计地区生产总值实现1078亿元，增长7.5%；全口径财政收入实现124亿元，增长7.5%，其中地方级财政收入实现82.5亿元，增长11.5%；固定资产投资完成938亿元，增长17.1%；社会消费品零售总额实现437.5亿元，增长12.5%。

　　——民生福祉继续增进、惠及城乡。预计城镇和农村常住居民人均可支配收入分别增长8%和11%。社保、教育、医疗等民生支出达到77.6亿元，占公共预算支出的36.7%。居民消费价格涨幅控制在2%。

　　——各项事业稳步发展、成果丰硕。改革开放全面深化，新型城镇化和生态文明建设扎实推进，区域交通枢纽地位进一步提升，基础设施加快完善，社会各项事业全面发展。

　　各位代表！面对2014年经济下行压力加大的复杂形势，面对极其特殊的发展环境和不利影响，我们坚决贯彻市委决策部署，着眼当前，考虑长远，抢抓机遇，创新突破，全力以赴稳增长、调结构、强基础、促改革、保生态、惠民生、谋发展，有力有序有效推进各项工作，实现了全市大局稳定和经济社会持续健康发展。主要做了以下工作：

　　一是着力抓好项目建设和招商引资，产业结构调整取得新进展。狠抓投资不放松，突出扩大增量、优化总量，大力提升发展质量和效益，不仅稳定了当期增长，也为长远发展打下了基础。全市开工3000万元以上项目1192个，其中亿元以上项目350个、10亿元以上项目30个，新上项目、工业项目比重分别达到83%和62%。四长医药工业园一期、东宝人胰岛素注射剂三期、金马异地新建等112个亿元以上项目如期竣工。调整完善招商政策，引进域外资金712亿元，实际利用外资3.8亿美元，引进亿元以上项目333个。广东恒大、上海中暨、江西仁和、中兴恒和、晋商联盟、台湾联华等知名企业落户通化。抢抓新一轮振兴东北机遇，积极谋划包装项目，生物医药集聚区建设通过国家评审，二道江区列入国家首批东北地区独立工矿区搬迁改造规划，已有19个重大项目和工程通过省发改委评审上报国家争取支持。争取国家和省项目资金43.8亿元。

　　工业支撑有力。组建联系服务企业工作队，全面加强产需衔接、银企对接和要素保障，千方百计稳工业、兴产业。规模以上工业实现产值1890亿元，增长14%，增速居全省前列。深入实施产业跃升计划，八大重点工业产业产值增长15%。医药产业产值突破千亿大关，达到1003亿元，增长23%。累计63户医药企业通过新版GMP认证。通钢进入国家行业准入名单，环保控制区拆迁安置取得突破性进展，新2号高炉正式投产，冶金产业亏损态势在逐步扭转。食品、建材、机械制造产业快速发展，能源、化工产业稳定回升。鸭绿江河谷葡萄酒庄及研发中心等葡萄酒产业项目开工建设，山葡萄栽培示范区成为国家级农业标准化示范区，葡萄酒产业产值增长16.9%。医药高新区被科技部认定为全省唯一创新型产业集群培育试点，医药等6个产业集群列入全省特色工业园区重点产业集群，省规划核准的7个特色工业园区新入驻企业20户。规模以上工业企业新增10户，总数达到548户。新创5件中国驰名商标。

　　服务业繁荣发展。召开了全市旅游产业发展大会，启动了金川国际旅游度假区、罗通山文化旅游度假区、万亩油菜花海等21个景区景点项目，开通了集安赴朝鲜1日、5日游，大泉源历史文化旅游区、通天山葡萄酒文化科技产业园、振国健康养生谷晋升为4A级景区，接待国内外游客增长25.6%，旅游收入增长35%。市区农产品批发市场投入使用，全省首家人参电子交易平台上线运营，淘宝网注册电商达到8700户、新增3800户。通化海关开关。外贸进出口总额实现5.8亿美元，外贸出口增长50%。

　　二是着力加快农业农村发展，县域经济转型升级取得新成效。实施农作物高光效栽培示范面积22.2万亩，建设万亩高产示范区40个，农业大户、专业合作社、家庭农场快速增加。粮食生产战胜夏季旱灾再获丰收，总产量达到39亿斤。建成标准化牧业小区210个，畜牧业产值增长20%。新建高标准基本农田16.2万亩，新增棚膜蔬菜面积4020亩。省级以上产业化龙头企业发展到54家，农产品加工量达到222万吨。国家有机食品基地发展到6个，绿色有机无公害农产品增加到330种。人参国际标准科技联盟在我市成立，紫鑫、百泉等人参精深加工项目投产，研发人参新资源食品179种，获批长白山人参品牌数量居全省第一位，人参产业产值突破100亿元。完成江河堤防、水库除险加固等水利工程73项。

　　县域经济竞相发展。梅河口市扎实推进扩权强县改革试点，经济增速和质量效益居全省县域前列。集安市狠抓旅游、参药等支柱产业创新发展，城市知名度进一步提升。辉南县推动特色产业集群发展，被命名为中国野生山核桃之乡、长白山林蛙之乡。柳河县城镇化建设取得新成效，获全国农业标准化示范县等多项殊荣。通化县生物医药产业迅猛发展，创建国家级生态县通过环保部现场核查。东昌区项目引进、企业重组力度加大，民营经济对财政的贡献率超过90%。二道江区强力推进经济转型，要素集聚能力和发展后劲明显增强。有6个县（市、区）全口径财政收入突破10亿元。在全省县域综合发展考评排序中，梅河口位列第1，通化县、集安、柳河分别位列第7、第12和第13，我市获组织推动奖。

　　三是着力改善城市承载功能，基础设施和城市建设取得新突破。通梅高速公路完成工程量的80%，通靖、通集、辉白高速公路和市区绕越线五月花互通工程开工建设。通化机场正式通航。市区改造各类棚户区131万平方米，配套完成老旧住宅小区环境综合整治15万平方米；滨江东路、西路全线贯通；江南新桥建成通车；街路巷路升级改造、绿化美化亮化等工程按期完工；新建换热站6座，铺设改造供热管网55公里，新楼入网供热面积110万平方米；综合改造二次供水泵房76座，解决了6.45万户居民用水和水质安全问题。改造地下供水管线104.7公里；新增煤气用户9634户；更新公交车50辆，新建公共候车亭100座，新开公交线路4条；城市管理、交通管理得到加强，物业管理覆盖率达到60%。收储土地563.4公顷，招拍挂供应土地267.9公顷。编制完成特色城镇化发展战略规划，城港经济带建设积极推进，县城和示范镇、重点镇建设力度加大。“智慧城市”建设全面启动。

　　四是着力推动改革创新，发展活力和动力得到新增强。政府机构改革顺利完成，食药监、工商、质监划归市属管理。事业单位分类工作基本完成。减少行政审批项目119项，政务大厅即办件达到97.6%。工商登记制度改革全面实施，新登记内资企业增长36%、个体工商户增长10.2%。民营经济主营业务收入增长15%，民营企业数量突破1万户。农村土地承包经营权确权试点稳步推进，土地流转比例达到29%。通化农村商业银行组建成立，新增小额贷款公司7家、村镇银行3家，土地收益保证贷款试点拓展到3个县区。4户企业在上海股交中心挂牌融资。在全省率先公开政府预算、部门预算和“三公”经费预算。组建了信通投资控股公司。获批国家和省科技专项56项，35项科研成果分获省市科技进步奖，各类创新型企业达到93户。成功举办了长白山现代中医药产业发展论坛、医药健康产业科技成果对接会，科技创业服务中心在孵企业数量成倍增长。与国内多所高校和科研院所签订战略合作协议，建成吉林农业大学、中科院长春应化所产学研用合作基地，科技成果交易中心投入运营。国家知识产权示范城市建设积极推进，在全省率先开展知识产权抵押融资贷款，专利申请量和授权量居全省前列。

　　五是着力推进绿色发展，生态环境保护建设迈出新步伐。在全省率先召开了生态环境保护建设工作会议，提升生态文明水平各项任务全面整体推进。组织开展生态环境专项整治，初步划定生态保护红线678平方公里。植树造林31.2万亩，清收林地85.4万亩，创建绿美示范村屯59个、森林小镇7个，查处涉林案件667起，实现了连续60年无重大森林火灾。哈泥河水源保护力度加大，龙湾群国家级森林公园被选入首批全球最佳保护地绿色名录。完成污染物减排和重点流域污染防治项目62个，市区空气环境质量新标准优良天数达到312天。249个农村环境连片整治项目通过验收。一批地质灾害整治工程顺利竣工。新增国家级生态乡镇9个、省级生态乡镇7个、省级生态村16个、市级生态村70个。我市被命名为省级园林城市、全国首批创建生态文明典范城市。

　　六是着力保障和改善民生，为群众办实事、解难题收获新成果。实施民生工作计划，120件实事如期完成。城镇新增就业4.1万人，农村劳动力转移就业29万人。养老、失业、医疗、工伤和生育保险新增参保4.7万人，企业退休人员月人均养老金增加154元，城市低保月标准、农村低保年标准分别提高到430元和2500元，新型农村合作医疗政府补贴人均增至320元，特困群体救助全面铺开。社区管理岗位工资人均增加1000元。市区保障性安居工程开工1.9万套，建设公共租赁住房3.8万平方米，1.7万户住房困难家庭享受到租赁补贴。市直行政事业单位职工住房公积金缴存比例成倍提高。改造农村危房5184户。建成农村饮水安全工程141处，8万农村人口受益。出台加快养老服务业发展实施意见，新建了24个社区老年人日间照料站。

　　社会事业全面进步。新建改建公办幼儿园8所，改造农村薄弱校25所，完成十三中、二十一中校舍新建，义务教育阶段中小学生人均公用经费城市上调60元、农村上调100元，职业教育得到加强。启动县级公立医院改革试点，基层医疗机构全部推行基本药物制度，传染病防治成效显著。市中心医院外科大楼和结核病防治所业务用房投入使用，完成216所标准化村卫生室建设任务。市科技文化活动中心落成，27个乡镇综合文化站达到国家规定标准。文艺创作取得新成果，群众文化活动丰富多彩，历史文化遗产保护利用不断加强。文化创意产业园建成运营。西山体育场改建工程竣工。在十七届省运会上取得好成绩。市残疾人康复中心被国务院残工委授予“残疾人之家”荣誉称号。民族团结进步和人口计生、广播电视、妇女儿童等事业实现新发展。

　　七是着力加强政风建设，行政效能和社会治理水平得到新提升。扎实开展群众路线教育实践活动，以“钉钉子”精神集中解决“四风”突出问题，会议、文件数量分别削减21%和18%，清理超标公车608辆、办公用房9.2万平方米，一批作风顽症和群众反映强烈的问题得到整治。严格依法行政，行政监察、审计监督取得新进展。自觉接受人大及其常委会的法律监督、工作监督和政协民主监督，办理人大代表建议120件、政协委员提案180件。“六五”普法成效明显。坚决查处违法违纪案件，立案399件，党纪政纪处分393人，移送司法机关26人。

　　社会保持和谐稳定。持续开展安全隐患排查整改，事故起数和死亡人数实现“双下降”，被评为全国安全生产月活动先进单位。排查调处矛盾纠纷1.3万件，一批信访积案得到解决。市长公开电话受理群众来电5.6万件。深入推进平安通化建设，严打整治成果突出，社会治安防控体系建设成效显著，科技管边控边能力明显提升。食品药品质量安全监管力度加大。国防动员和双拥共建工作扎实推进。国家安全、外事侨务、气象、地震监测等工作都取得了新成绩。

　　各位代表！回顾一年来的工作，我们深感成绩来之不易！这是市委统揽全局、科学决策的结果，是市人大、市政协有效监督、大力支持的结果，是全市人民发挥聪明才智、团结奋进的结果。在此，我代表市政府，向全市人民，向人大代表、政协委员，向各民主党派、工商联和无党派人士、人民团体，向驻通部队、武警官兵，向驻通国省属企事业单位，向关心和支持通化改革发展的同志们、朋友们，表示衷心的感谢！

　　在肯定成绩的同时，我们也清醒地看到，前进道路上还有不少困难和问题：经济发展中长期积累的体制性、结构性难题还没有从根本上得到破解，产业层次不高，服务业发展相对滞后，大项目支撑不足，科技创新能力不强，调结构、转方式的任务十分艰巨；市政基础设施薄弱，地下管网陈旧老化严重；创新社会治理面临许多新情况新问题，化解社会矛盾难度加大，不稳定因素仍然较多，安全发展形势不容乐观，一些群众的利益诉求尚未很好解决，维护社会和谐稳定的任务还很繁重；个别公职人员缺乏责任感和担当精神，有的“为官不为”，违法违纪案件仍有发生。这些问题，需要我们采取有效措施，认真加以解决。

　　二、2015年工作安排

　　2015年是全面完成“十二五”规划的收官之年，也是我市发展在关键节点谋求突破的重要一年。国家明确提出支持东北新一轮振兴发展、加快沿边地区开发开放等一系列重大举措，全省推进东部地区绿色转型发展，为我们加快全面振兴提供了难得机遇。我市生态环境优良，是我省东部物种资源最为丰富、最具代表性的地区，资源利用转化潜力巨大；区位优势已经凸显，正成为东北东部重要的交通枢纽、商贸中心、物流集散地和吉林省最具现实意义的入关出海窗口；产业特色鲜明，产业转型升级基础坚实、空间广阔。我们要找准和发挥自身优势，以强烈的责任意识和担当精神，抢抓机遇，乘势而上，努力在新一轮振兴发展中开创新局面、迈上新台阶。

　　2015年，市政府工作的总体要求是，认真贯彻党的十八届四中全会和中央经济工作会议、省委十届四次全会和全省经济工作会议精神，认真落实市委六届四次全会部署要求，抢抓新一轮振兴东北机遇，坚持稳中求进、改革创新，坚持绿色转型、全面振兴，坚持依法行政、建设法治政府，全面提升发展质量和效益，精心描绘好“十三五”发展蓝图，努力让城乡居民生活得更加美好！

　　主要预期目标：地区生产总值增长8%左右，财政收入增长、城乡居民收入增长与经济发展同步，固定资产投资增长15%以上，社会消费品零售总额增长12%以上，居民消费价格涨幅控制在3%以内，城镇登记失业率控制在4.5%以内，节能减排完成省下达的任务指标。

　　（一）突出抓好投资拉动、项目带动。围绕重点产业、基础设施、改善民生、社会建设和生态环保“五大板块”，新建续建3000万元以上项目1200个、亿元以上项目360个、10亿元以上项目32个，固定资产投资达到1100亿元。开工建设四环100万套电子医疗器械、万通40亿片感通片等新项目，加快实施通化生物科技产业园、医药高新区新材料产业园等续建项目，推动东宝120万瓶人血清白蛋白注射剂、联合260亿粒药用胶囊等项目建成投产。强化项目前期工作，注重规划、环保和集约节约用地，提高投资强度和产出效益。鼓励民间资本进入基础产业、基础设施和公用事业等领域，力争民间投资所占比重达到80%。

　　主动适应经济发展新常态，把握战略机遇，加强战略研究，挖掘资源潜力，选准功能定位，做好“十三五”规划编制工作，在更高起点上谋划通化发展。着力谋划一批对经济发展、结构调整带动作用强的重大项目，对推进基础设施、新型城镇化、社会事业和社会管理建设、生态环保、改善民生作用显著的重大工程，为增后劲、上水平提供支撑。

　　（二）强力推动工业发展和产业转型升级。发挥工业资源、资产优势，制定“巨人”企业培育计划，推进现有企业跨区域战略联合重组，支持骨干企业改建扩建和优势产品产业化扩能改造，做大做强本土企业和实体经济。重视和支持中小微企业发展，进一步减轻企业负担，帮助企业解决融资、市场和技术创新、人才招引等问题。滚动实施15个工业化与信息化融合应用项目，拓展信息技术在产品研发、生产、营销、配送和售后服务等环节的集成应用。深入实施质量兴市战略，新创一批中国驰名商标、名牌产品和地理标志保护品种。技改投资增长12%，新产品产值率达到10%。规模以上工业产值增长10%以上。新增规模工业企业10户以上。

　　着力推进支柱优势产业提升、特色资源产业发展和战略性新兴产业培育，努力形成创新发展、多元支撑的产业格局。放大国家医药高新区的政策集成和省部共建机制优势，创办长白山医药健康产业论坛，搭建公共创新服务、中试转化、医药物联网系统等功能完善的综合服务平台，突出发展现代中药、生物制药、保健和功能食品，积极发展医药商业与流通、医药包装、医疗器械、中药材规范化生产、医疗与健康服务，全面提升现代医药城整体功能。启动新一轮医药健康和生物医药产业布局，创建全省医药健康产业示范区，争取生物医药集聚区列入国家试点。全面完成医药企业新版GMP改造。支持服务通钢建设冶金产业园，调整产品结构、开发高端品种钢，积极为一汽、油田等大企业配套，逐步实现转型发展；大力发展通钢协作配套产业，建设一批钢材深加工项目，提高通钢产品就地转化率和制品化率，促进冶金产业企稳回升。深度开发长白山特色食品资源，抓好长流食品加工产业园等40个超亿元项目，做大林蛙、山珍、畜禽、白酒、葡萄酒、矿泉水等产品规模，提升绿色食品产业发展水平。加快鸭绿江河谷葡萄产业带建设，筹建通葡酒庄及葡萄酒文化主题公园，促进通化葡萄酒品牌振兴。推进江东老工业区搬迁改造，规划建设现代装备制造产业园，石油装备扩能改造工程建成投产。抓好玄武岩产业集聚区建设和高端后续项目开发，搞好石墨、钾长石、火山岩等资源综合开发，积极推进柳河、通化县、集安石油项目勘探，培育壮大新材料、新能源、节能环保等战略性新兴产业。开展“食品药品生产质量管理提升年”活动，加强质量安全监管，打造药品食品等生产信用氛围，确保支柱优势产业诚信健康发展。

　　推动服务业提速增效。升级发展商贸物流业，启动建设物流产业园、木材物流园区和现代农副产品交易中心，加快欧亚、义乌、中东等商圈建设，争取更多的新兴业态和知名品牌进驻通化。争创国家“智慧城市”试点，规划建设“北斗数谷、智慧通化”项目，逐步实施城市公共信息平台、综合管理系统、智慧旅游、智慧交通、智慧教育、智慧医疗、信息惠民等应用项目。加快建设旅游支柱产业，全力打造吉林省生态文化旅游第二极和东北亚国际重要旅游目的地。实施长鹿国际休闲游博园、龙溪健康养生谷等重点旅游项目，启动建设旅游集散中心，发展沟域旅游、乡村旅游、冰雪旅游和红色旅游，开发森林、湿地旅游和鸭绿江河谷观光游。开展特色景区评选活动，争取高句丽遗迹、龙湾国家森林公园晋升5A级景区，推动龙湾野生杜鹃花卉旅游节、鸭绿江国际枫叶旅游节等节庆活动形成知名品牌。加大宣传推介力度，扩大通化旅游的知名度、美誉度。规划建设英额布、湾湾川、白鸡峰等国际养生休闲度假基地，加快发展高端养老养生产业。系统谋划以中药培植、医药健康、绿色食品、保健养生、避暑度假、休闲旅游为主的大健康产业发展。发展文化产业，加快建设文化产业示范基地，推进松花石资源产业化综合开发利用，促进文化与旅游、商贸、养生保健等产业融合发展。

　　（三）加快县域和农业农村经济发展。建立统筹布局、区域协调、资源共享、优势互补的市县关系，助推各县（市、区）科学谋划城镇和区域发展定位，培育壮大特色产业集群。优化种植结构，集成推广保护性耕作、高光效栽培等技术措施，重大粮食增产技术推广面积达到300万亩以上。抓好国家农业科技园区建设，统筹规划黄金水稻带，搞好东昌和二道江环城农业产业带、集安特色农业示范区、辉南山核桃之乡、柳河生态农业产业园、通化县有机食品科技园等项目实施。抓好20项农业产业化重点项目。建设200个标准化牧业小区，规模化养殖比重达到85%以上。实施农特产业基地建设工程，每个县（市、区）新建1个以上示范基地。新建恒大人参产业园等超亿元项目，完善人参电子交易服务平台，开发人参新资源食品，人参产业产值增长20%以上。开工建设长白山林蛙产业园，加快发展林蛙系列产品。树立循环经济理念，调整单一生产籽粒的农作物种植格局，新上90万吨玉米秸秆压缩饲料等项目，推进农作物秸秆资源化综合利用。依托丰富的菌基资源，推动食（药）用菌产业快速发展，食（药）用菌生产面积达到500万平方米。大力实施“菜篮子”工程，进一步扩大棚膜蔬菜种植面积。培植、扶持、做大特优农特产品品牌，新创绿色有机无公害农产品标识20个以上。继续推进新农村建设，完成45个省级重点村建设任务。

　　（四）积极稳妥推进新型城镇化。坚持规划先行，完成城镇总体规划及中心城市分区规划编制工作，制定出台空间共享、招商受益、有效扶持的政策，明确城镇化长远发展方向和重点建设任务，构建特色鲜明、布局合理、功能完善的现代城镇体系。推动中心城市率先突破，优化城市发展布局结构，提高空间资源利用效率。加快城港经济带建设，实施区域专项规划和产业园区规划，抓好二密、三源浦两个起步区的基础设施建设和项目谋划引进，增强集聚带动功能。推动湾湾川生态新城规划建设。加快县城提质扩容和工业重镇、商贸大镇、旅游名镇建设，推进金厂镇省级城镇化试点和朝辉（朝阳镇、辉南镇）一体化发展试点，在各县（市、区）各确定1个示范镇、2个重点镇先行先试。规划建设朝鲜族、满族特色民俗村，发展有历史记忆、区域特色、民族风情的特色城镇。培育城镇特色产业，强化资源环境支撑，加强水、电、气、路等基础设施建设，改善教育、医疗、文体等公共服务条件，提高城镇化质量。

　　本着重基础、重民生、重生态、重文化、重品质的原则，高起点规划城市，高水平建设城市，高效能管理城市。市区城建计划投资50亿元。加快城市棚户区和工矿棚户区改造，开工建筑面积100万平方米，推动改造向周边拓展。综合整治老旧住宅小区15万平方米，改善环境设施。升级改造城区9条主次街路，拓宽改造部分街路路口。新建续建江南新区9条道路，开工建设江南地下商业街人防工程，建成联结欧亚和中东的地下通道及商圈。新建佟佳江景观公园，搞好滨江沿河、重点街路绿化美化。开展城市地下管网普查，加快老旧地下管网改造，努力完成老旧燃气管网改造，提升城市安全保障能力。继续实施二次供水改造，改造16个区域二次供水设施，解决3万户居民用水和水质安全问题。改造地下供水管网40公里。完成热源厂扩容改造，新建换热站8座，铺设供热管网14公里。基本完成“气化通化”工程。

　　深入推进国家卫生城市、园林城市、森林城市和全国文明城市“四城”联创，全面加强城市管理。巩固提升主街主路保洁水平，强化城市进出口、滨江沿河、背街小巷、城乡结合部等区域的环境综合整治，强化房屋安全和质量管理，实现违法建设“减存量、零增量”。坚持政府推动、社会参与和市场运作“三位一体”，研究制定城市经营管理政策措施，加快“以城养城、以城兴城”机制建设。大力实施市民素质教育提升工程，调动广大市民参与城市管理的积极性、主动性。

　　（五）深化改革、扩大开放。按照国家和省里的顶层设计，从市情实际出发，积极稳妥推进深化改革各项工作。建立行政审批项目动态清理调整机制，开通审批项目管理平台。对保留的审批事项，严格固化收费标准，精简审批环节，优化审批流程，压缩审批时限，形成规范高效的审批运行机制。深化事业单位分类改革，推动公益服务类事业单位去行政化。继续推进工商登记制度改革，健全市场准入制度，加强市场监管，促进中介组织依法依规经营。建立社会资本参与市政基础设施建设项目运营库。落实突出发展民营经济各项政策，鼓励民营企业建立现代企业制度，加强创业服务中心和孵化基地建设，健全中小民营企业信用体系、融资担保机制。民营经济主营业务收入增长10%。统筹推进财税、土地、公共资源交易、价格等改革。加强政府性债务管控，完善债务风险预警及化解机制。规范信通投资控股公司运营管理。深入开展金融创新，大力发展产业金融，积极引进股份制银行、民营银行在我市设立分支机构。金融机构新增放款力争达到100亿元。发展保险业，推动保险产品创新。抓好企业上市，争取4户企业在上海股交中心和“新三板”挂牌融资。加快构建新型农业经营体系，促进土地经营权有序流转，支持发展家庭农场、种养大户、专业合作社和产业化龙头企业等新型主体。推进农村集体经营性建设用地出让、租赁、入股试点。深化户籍制度改革，完善新型城镇化配套政策。建立健全科技成果产业化推进机制，统筹城乡义务教育资源均衡配置，深化基本药物制度和基层医疗卫生机构综合改革，支持社会资本参与文化产业发展。创新有效预防和化解矛盾体制，促进新形势下社会组织健康发展。

　　提升开放发展水平。发挥好入关出海门户城市的特殊功能作用，努力把我市打造成为面向东北亚开发开放的前沿城市。加快通丹经济带规划建设，深度融入辽宁沿海经济带和环渤海经济圈，积极推动东北东部经济带上升为国家战略。加快鸭绿江中朝经济合作带建设，争取国家尽早批复建设集安跨境经贸合作区，推进界河大桥联运通行。加快开放平台载体建设，促进各类开发区转型升级、创新发展。医药高新区地区生产总值增长20%以上，开工、投产超亿元项目30个以上。各县（市、区）开发区都要培育集聚效应明显的产业集群。策划打造国际文化交流平台，积极发展友好城市。加强出口基地建设，优化海关通关服务，促进外贸稳定增长。坚持引资与引技、引智并举，创新招商引资方式，强化与国内外重点地区产业对接互动，引进更多的知名企业和战略投资者，促进合作项目落地，推动落户企业再投资。规划建设中韩产业园、台湾生物科技产业园。

　　（六）深入实施创新驱动战略。推进以企业为主体、以市场为导向、以高校院所为依托的产业技术创新战略联盟建设，提升原始创新、集成创新、引进消化吸收再创新能力。争取建设国家自主创新示范区，突出抓好重大科技攻关转化项目，重点策划推进生物医药、现代中药、新材料三大领域的创新发展，抢占未来发展制高点。完成百户创新企业集群工程，国家级高新技术企业达到35户、省级创新型和科技型企业达到65户。申报国家和省科技专项60项，争取大株红景天注射液等15个项目进入全省“双十”工程和省级以上重大专项。积极搭建食品药品网上经营平台，推进医药企业新型决算试点，建立国家新政策辅导机制。大力推动与高等院校和科研院所的合作交流、项目对接，加强产学研用协同创新，加大研发团队引进力度，加快建设科技成果交易中心、院士工作站和科技企业孵化器，促进科研院校科技成果优先在我市转化。建成国家知识产权示范城市。实施人才培养引进计划，培养技术技能型、实用型人才，引进专家型、“候鸟型”人才，鼓励通化籍高校毕业生回乡创业就业。

　　（七）加强基础设施和生态文明建设。实现通梅高速公路竣工通车，加快建设通靖、通集、辉白高速公路和通化大街，开工建设西昌立交互通工程，建成五月花立交互通工程。推进建设四平至通化至松江河快速铁路，筹划通化火车站改造，筹建公路客运换乘中心。完善通化机场及周边配套设施，积极申请口岸机场，研究开发国际航线和低空旅游飞行项目。推动建设通丹陆港。实施大中型电网和农网改造升级工程。抓好江河堤防、水库除险加固等61项水利工程。推进城市供水水源综合改造，筹建第二净水厂和应急水源。

　　加快建设山清水秀、生态宜居的美丽通化。坚持严格保护、科学开发、创新提升、整体跨越，在保护中发展，在发展中保护，让通化的天更蓝、山更绿、水更清。制定生态环境保护建设总体规划，全面划定和严守生态保护红线，健全资源有偿使用和生态补偿赔偿制度。推进蓝天工程，落实大气污染防治计划，加强空气质量监测预警，提高排放准入门槛；抓好冶金、火电等高耗能行业技术升级、节能改造，推动燃煤机组脱硫脱硝改造，严格废弃物焚烧管理和扬尘污染治理，渐次淘汰黄标车并严禁转入落籍。实施青山修复工程，推动25度以上坡耕地退耕还林，因地制宜发展林药、红松果林、山核桃、大榛子、林下参等林下经济，推广沟域生态经济模式；依法开展清收还林，清收林地11万亩，还林4万亩，力争两年任务一年完成；造林绿化10.4万亩，标准化绿化村屯35个；禁伐红松、核桃楸等天然果林；确保无重大森林火灾；治理水土流失面积240平方公里；加快集梅公路沿线裸露山体生态修复，提升龙湾、五女峰、石湖、白鸡峰等国家级森林公园生态功能；规划建设长白山药用植物园和药用资源保护区。抓好碧水工程，实施最严格的水资源管理制度，加快哈泥河流域等饮用水源的生态环境综合治理，完成重点流域16个污染防治项目，打击违法排污、破坏生态行为；加强森林、湿地自然保护区建设，抓好国家级自然保护区晋级申报工作，对保护区重要区域实施生态移民工程；推进穿城水系和水域景观提升工程，规划建设以湿地公园、观光廊道、生态小镇和农家乐为重点的生态带、景观带。筹划市区南山生态移民工程，推动玉带河水质达标，搞好周山沟河及公路、铁路沿线环境治理。加快建设市区垃圾焚烧发电工程。启动农村人居环境改善“六大工程”，争取省里在我市开展农村环境连片整治整体推进试点。打好土壤污染防治攻坚战。推进二道江矿山地质环境综合整治二期工程，搞好尾矿库、老矿山和地质灾害普查治理。抓好国家级生态市创建工作，创建省级生态村12个、生态乡镇5个，争创柳河省级生态县。争取开展生态系统生产总值（GEP）核算试点。

　　（八）协调发展各项社会事业。加强素质教育，促进义务教育均衡发展。实施幼儿教育覆盖工程，继续推进农村薄弱校改造，实施市一中校舍扩建和中小学示范性综合实践基地等项目。加快发展现代职业教育，深化产教融合、校企合作。推动中职与高职教育衔接，支持通化师范学院加快发展高等职业教育。加强教育信息化建设。整合优化医疗卫生资源，促进医疗事业快速发展。筹划市妇幼保健院新建、市人民医院扩建等工程，提高社区卫生服务中心和基层医疗机构服务水平，完成249个村卫生室标准化建设。加强重大传染病防控。坚持计划生育基本国策，落实单独两孩生育政策。挖掘开发文化资源，创建城市文化品牌，提升城市文化软实力。繁荣文艺创作，打造艺术精品。完善公共文化服务体系，推进图书馆和社区文化活动室、乡镇文化站建设。加强历史文化遗产保护。建成市全民健身中心，建设和完善城区健身路径和农村文体广场。大力推进民族团结进步事业，深入实施兴边富民行动。依法加强宗教事务管理。保障妇女儿童权益，发展老龄、残疾人事业。支持国防和驻通部队建设，推动军民融合深度发展，争创全国双拥模范城“八连冠”。

　　（九）统筹做好保障和改善民生工作。组织实施好市委确定的“十大民生工程”，集中力量办100件左右民生实事。城镇新增就业3.9万人，农村劳动力转移就业23.5万人。加快创业园和创业培训基地建设，开展订单、定向等多种形式职业技能培训。按政策提高基本养老金、失业保险金、城乡低保、城镇居民医保、新农合补助标准和低保对象大病医疗救助标准，稳步提高城乡养老保险参保率。完善困难群众救助帮扶长效机制，在基本生活、医疗、就学、住房等方面加大政策优惠和扶持力度。大力发展养老产业，抓好养老服务设施和居家养老等网络建设，规划建设养老服务中心，完成东昌区省级养老服务试点。优化社区管理资源，健全社区服务体系。加快保障性安居工程建设，为1.6万户低收入住房困难家庭发放住房租赁补贴。全面加强物业管理，尽快提高基本物业服务覆盖率。抓好社保资金、住房公积金和房屋维修基金管理。更新城市公交车30辆，科学设置公交线路。加快建设停车场，强力整治交通秩序，统筹解决停车难和高峰拥堵问题。积极推进城中村改造，通过几年的努力完成全市32个城中村改造任务。改造农村危旧房5000户。建成农村饮水安全工程126处，解决5万农村人口饮水安全问题。

　　全力维护社会稳定。深入推进安全发展，认真贯彻新的《安全生产法》，严格落实安全生产长效机制和主体责任，持续开展生产安全、消防安全、道路交通等各领域安全监管，强化老旧燃气管网巡查检查，整改隐患、不留死角，确保生产安全、人民平安。加强食品药品安全监管，保障群众饮食用药安全。加大信访工作力度，切实解决群众的合理诉求。深入推进社会治安防控体系建设，深化平安社区、平安村屯创建，健全覆盖城乡的公共视频监控网络，严厉打击各类犯罪，建设平安通化。健全应急管理机制，完善专项应急处置措施。强化党政军警民合力治边，维护边境稳定和国家安全。

　　三、加强法治政府建设

　　认真落实市委关于全面推进依法治市的部署要求，深入推进依法行政，全力打造职能科学、权责法定、执法严明、公开公正、廉洁高效、守法诚信的法治政府。

　　牢固树立法治理念。坚持学法、遵法、守法、用法，提高运用法治思维和法治方式深化改革、推动发展、化解矛盾、维护稳定的能力。坚定法治信仰，增强法治观念，尊重法律权威，真正使遵纪守法成为一种习惯、恪守法律规则成为一种常态。自觉接受党内纪律监督、人大法律监督和工作监督、政协民主监督、社会舆论监督，深入推进“五权”工作，防止权力滥用。坚持依法办事，严格规范公正文明执法，加强普法教育，引导全民自觉守法、遇事找法、解决问题靠法。

　　依法全面履行职能。深化政府机构改革，推进组织机构、职能配置、运行方式法定化。实施简政放权，进一步减少、下放行政审批事项，做到放得下、接得住、管得好。制定权力清单，依法公开权力运行流程，规范行政执法自由裁量权范围、种类和幅度，坚持法无授权不可为。建立责任清单，厘清部门职责边界，明确岗位责任，做到法定职责必须为。推进权责一致、责任和财力保障相匹配的新型事权管理体制。实行负面清单，激发市场主体活力，实现法无禁止皆可为。坚决查纠不作为、乱作为，坚决克服懒政、怠政，坚决惩处失职、渎职。

　　健全依法决策机制。健全重大问题公众参与、专家论证、风险评估、合法性审查和集体讨论决策机制，明确重大决策范围、审查重点和决定程序，进一步提高政府公信力和执行力，防止拍脑袋决策和违法决策。探索重大决策终身责任追究及责任倒查机制，减少决策失误，提高决策质量。普遍推行政府法律顾问和决策咨询制度，有效利用“外脑”提升决策信息和智力支撑水平。完善政府规范性文件制定程序，做到重大决策于法有据。

　　深化执法体制改革。完善行政执法体系，强化行政效能，降低行政成本，推进食品药品安全、城市建设管理等领域综合执法，解决权责交叉、多头执法问题。落实行政执法责任制，加快研究制定重大执法决定审核制度和执法全过程记录制度，解决有法不依、执法不严、违法不究甚至以权压法、徇私枉法问题。强化监管职责，优化资源配置，充实基层一线行政执法力量，推动执法监管制度化、规范化、程序化。加大政务公开力度，推进决策公开、执行公开、管理公开、服务公开、结果公开，加强电子政务和政府网站建设，让行政权力在阳光下运行。

　　各位代表！我们面临着经济发展新常态，必须认识新常态，适应新常态，全面加强政府自身建设，增强引领新常态的本领，努力实现振兴发展新作为。

　　我们一定坚持为民执政。牢记全心全意为人民服务根本宗旨，始终把人民利益放在第一位，时刻与群众保持血肉联系，坚持不懈地为群众办实事、解难题、谋福祉，让振兴发展成果更多、更公平地惠及全市人民。

　　我们一定坚持高效理政。咬定目标不放松，只争朝夕、敬终如始、善做善成，一张蓝图干到底。上级决策的，要不折不扣地贯彻；部署了的，要不厌其烦地督导；督导了的，要既看过程、更看结果，真正实现“政无戏言”。

　　我们一定坚持干事勤政。自觉践行“三严三实”要求，坚持打基础、利长远，坚韧不拔、夙夜在公，脚踏实地、主动作为，勤勉尽责、争创一流，推动各项工作与时俱进、创新发展。

　　我们一定坚持从严治政。严格执行中央八项规定和省委、市委具体规定，持之以恒狠刹“四风”，勤俭办一切事情，巩固拓展教育实践活动成果。认真落实党风廉政建设责任制，全面推进惩治和预防腐败体系建设，加强行政监察、审计监督，严肃查处违纪违法案件，全面塑造为民务实清廉的政府形象。

　　各位代表！勤劳智慧的通化人民，曾经创造过许多历史辉煌，在新一轮振兴发展的征程中，一定能谱写出新的壮丽篇章。让我们在市委的坚强领导下，抢抓机遇，敢于担当，重行务实，真抓实干，为实现通化全面振兴，让城乡居民生活得更加美好而努力奋斗。
